# Functional Analyses of Rare Germline Missense *BRCA1* Variants Located within and outside Protein Domains with Known Functions

**DOI:** 10.3390/genes14020262

**Published:** 2023-01-19

**Authors:** Henrikke Nilsen Hovland, Eunice Kabanyana Mchaina, Hildegunn Høberg-Vetti, Sarah Louise Ariansen, Wenche Sjursen, Marijke Van Ghelue, Bjørn Ivar Haukanes, Per Morten Knappskog, Ingvild Aukrust, Elisabet Ognedal

**Affiliations:** 1 Western Norway Familial Cancer Center, Haukeland University Hospital, 5021 Bergen, Norway; 2 Department of Medical Genetics, Haukeland University Hospital, 5021 Bergen, Norway; 3 Department of Clinical Science, University of Bergen, 5021 Bergen, Norway; 4 Faculty of Health Studies, VID Specialized University, 5009 Bergen, Norway; 5 Department of Medical Genetics, Oslo University Hospital, 0424 Oslo, Norway; 6 Department of Medical Genetics, St. Olavs University Hospital, 7006 Trondheim, Norway; 7 Department of Clinical and Molecular Medicine, Norwegian University of Science and Technology, 7491 Trondheim, Norway; 8 Department of Medical Genetics, University Hospital of North Norway, 9038 Tromsø, Norway; 9 Department of Clinical Science, UiT The Arctic University of Norway, 9019 Tromsø, Norway

**Keywords:** breast, ovarian, hereditary cancer, *BRCA1*, variants of uncertain significance, VUS, functional assays

## Abstract

The BRCA1 protein is implicated in numerous important cellular processes to prevent genomic instability and tumorigenesis, and pathogenic germline variants predispose carriers to hereditary breast and ovarian cancer (HBOC). Most functional studies of missense variants in *BRCA1* focus on variants located within the Really Interesting New Gene (RING), coiled-coil and BRCA1 C-terminal (BRCT) domains, and several missense variants in these regions have been shown to be pathogenic. However, the majority of these studies focus on domain specific assays, and have been performed using isolated protein domains and not the full-length BRCA1 protein. Furthermore, it has been suggested that *BRCA1* missense variants located outside domains with known function are of no functional importance, and could be classified as (likely) benign. However, very little is known about the role of the regions outside the well-established domains of BRCA1, and only a few functional studies of missense variants located within these regions have been published. In this study, we have, therefore, functionally evaluated the effect of 14 rare *BRCA1* missense variants considered to be of uncertain clinical significance, of which 13 are located outside the well-established domains and one within the RING domain. In order to investigate the hypothesis stating that most BRCA1 variants located outside the known protein domains are benign and of no functional importance, multiple protein assays including protein expression and stability, subcellular localisation and protein interactions have been performed, utilising the full-length protein to better mimic the native state of the protein. Two variants located outside the known domains (p.Met297Val and p.Asp1152Asn) and one variant within the RING domain (p.Leu52Phe) were found to make the BRCA1 protein more prone to proteasome-mediated degradation. In addition, two variants (p.Leu1439Phe and p.Gly890Arg) also located outside known domains were found to have reduced protein stability compared to the wild type protein. These findings indicate that variants located outside the RING, BRCT and coiled-coiled domains could also affect the BRCA1 protein function. For the nine remaining variants, no significant effects on BRCA1 protein functions were observed. Based on this, a reclassification of seven variants from VUS to likely benign could be suggested.

## 1. Introduction

Through interaction with myriad protein partners, the multifunctional BRCA1 protein is involved in numerous important cellular processes to prevent genomic instability and tumorigenesis. While pathogenic germline alterations including missense variants in *BRCA1* predispose carriers to hereditary breast and ovarian cancer (HBOC), the role of variants of uncertain significance (VUSs) is unclear [1]. Rare missense variants constitute a major part of all *BRCA1* VUSs, and are particularly challenging to classify due to limited or conflicting evidence. 

**Figure 1:**
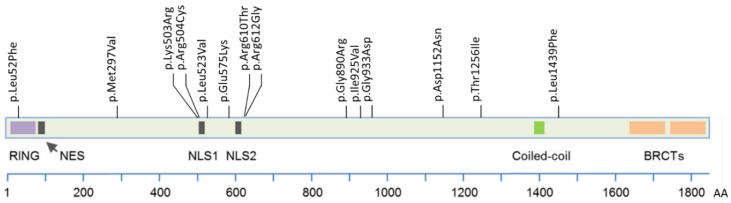
Schematic presentation of *BRCA1* and location of the investigated missense variants. RING = Really Interesting New Gene, NES = Nuclear Export Signal, NLS = Nuclear Localisation Signal, BRCT = BRCA1 C-terminal. Figure adapted from [8].

The *BRCA1* gene encodes a large protein of 220 kDa, primarily located in the nucleus, which consists of several functional domains (Figure 1). The N-terminal Really Interesting New Gene (RING) domain (aa 22–64) binds to BRCA1-Associated RING Domain protein 1 (BARD1), where heterodimerisation of the complex provides E3 ubiquitin ligase activity [2,3,4]. Two nuclear localisation sequences (NLS) (aa 503–508 and 607–614) allocate the BRCA1 protein to the nucleus where it exerts its functions. The coiled-coil domain (aa 1364–1437) located towards the C-terminal is involved in binding to the Partner and Localiser of BRCA2 (PALB2). Through the BRCA1 C-terminal (BRCT) domain (aa 1646–1736 and 1760–1855), BRCA1 interacts with multiple proteins involved in transcription and DNA damage response [5,6]. In addition to the established protein domains, BRCA1 contains an approximately 1500 residue unstructured central non-conserved region, of which very little is known [7]. 

Functional assays are considered as evidence of supportive to very strong strength for variant classification in the ACMG-AMP guidelines (BS3 or PS3 evidence) [9,10]. According to the *BRCA1* specific guideline for variant interpretation from CanVIG-UK, five functional protein studies are suggested with specific recommendations regarding the strength of their respective functional evidence [11,12,13,14,15,16]. However, only two of these studies use the full-length BRCA1 protein [11,12]. This is also the case for several other BRCA1 functional studies published to date, which focus primarily on variants located in the RING and BRCT domains using plasmid constructs expressing only parts of the full-length protein [17,18,19,20,21,22]. In addition, several of the previously published studies perform assays to study only one of the multiple functional characteristics of the BRCA1 protein separately, such as ubiquitination, transcriptional activation or homologous recombination repair (HRR). However, as *BRCA1* VUSs are distributed throughout the entire protein including regions outside well-established domains, examining only a single assay may be misleading [23]. Hence, to clarify how variants in the more non-conserved parts of the protein can potentially affect its functions, there is a need for several functional assays utilising the full-length protein to mimic the more native state of the BRCA1 protein. Some of the protein functions of BRCA1 also involve several domains of the protein, and consequently there is a need for multiple functional assays covering different activities. 

Several publications have suggested that most *BRCA1* missense substitutions located outside the well-established and conserved RING, coiled-coil and BRCT domains could be classified as (likely) benign, arguing that pathogenic missense variants are infrequent in these regions, which are thought to tolerate variations and be without essential functions [24,25,26]. In a recent publication, classification of *BRCA1* missense variants available in the public database ClinVar was used to illustrate this, and the authors suggest the incorporation of criteria regarding coldspots to improve the ACMG-AMP guidelines for *BRCA1* variant interpretation, as a counterweight to hotspots [27]. Noteworthily, a coldspot criterion is included in the BP1 evidence in the *BRCA1/BRCA2* gene-specific guidelines for variant interpretation from CanVIG-UK, which states that the location of a missense variant outside the RING, coiled-coil and BRCT domains is supporting evidence towards benign effect [28]. On the other hand, the approximately 1500 residue central region of BRCA1 has been suggested to act as a long flexible scaffold for intermolecular interactions which obtain a more ordered structure upon binding to protein partners, and may, thus, still be functionally important in the DNA damage response [7,29,30,31,32]. Furthermore, amino acid residues located outside well-established domains in the primary structure of the polypeptide chain can potentially interact with or become part of important structural and functional elements in the native folded three-dimensional structure of the BRCA1 protein. This indicates that replacing amino acid residues located outside an important protein domain could still possibly affect both the structure and function of the protein. 

The purpose of this study was, therefore, to functionally characterise a set of 14 *BRCA1* VUSs, of which 13 variants are located outside the known domains, by multiple different protein assays utilising the full-length BRCA1 protein. The *BRCA1* VUSs were selected from our recently published study of *BRCA1* variants detected in families with suspected HBOC in Norway, “*BRCA1* Norway” [33]. Since the majority of the VUSs investigated in this study are located outside the known protein domains of BRCA1, we aimed to use not only BRCA1 specific assays but also more general protein assays to assess their impact on protein expression, protein stability and subcellular localisation. Based on this, we wanted to investigate the hypothesis stating that *BRCA1* variants located outside the known protein domains are benign and of no functional importance. Furthermore, we aimed to use the data gathered from the different functional assays, in combination with other available information, as a tool to clarify the pathogenicity of these variants.

## 2. Materials and Methods

### 2.1. Plasmids and Construction of BRCA1 Variants

The plasmid pDEST-mCherry-LacR-BRCA1 encoding mCherry-tagged wild type (WT) full-length human BRCA1 protein was a gift from Daniel Durocher (Addgene plasmid #71115; http://n2t.net/addgene:71115 (accessed on 2 January 2020); RRID:Addgene_71115) [34]. This plasmid will hereafter be assigned *BRCA1* WT, or WT only. The *BRCA1* missense variants (listed in Supplementary Table S1) were introduced in the WT plasmid using the QuikChange II XL Site Directed Mutagenesis Kit (Agilent Technologies, Santa Clara, CA, USA). Primers used to produce variants of interest and control variants are available upon request. The empty vector (EV) plasmid pDEST-mCherry-LacR, hereafter assigned EV, was also kindly provided by Daniel Durocher [34]. All plasmids were prepared by QIAfilter Plasmid Maxi Kit (QIAGEN, Hilden, Germany), and the presence of the altered variants, in addition to the whole *BRCA1* insert, were verified by Sanger sequencing. The variants were all selected from our previous study, “*BRCA1* Norway”, and were reported as VUSs in ClinVar or classified as VUS by one or more of the Norwegian medical genetic departments at the time of selection [33]. Some of the variants were classified as both VUS and likely benign by different departments, and these were specifically included, aiming to harmonise the variant classification between the different departments. Intentionally, variants throughout the whole *BRCA1* gene were selected, and all variants except one (located within the RING domain) are located outside the well-established RING, coiled-coil and BRCT domains (Figure 1). For each assay, benign and pathogenic control variants were chosen. If possible, variants tested previously by the same type of assay were preferred as controls. A recurring issue and a limitation for all assays performed in this study was the lack of well-established relevant pathogenic missense control variants located in the regions outside of the known domains. No pathogenic variants outside of these regions were found in ClinVar or the literature. This made it difficult to fulfil the requirement of a sufficient number of control variants as suggested by Brnich et al. [10]. For investigations of co-immunoprecipitation assays with BARD1 and PALB2, controls were chosen from the relevant regions (RING and coiled-coiled domain, respectively).

In the co-immunoprecipitation assay, the plasmids pcDNA6.2-BARD1-V5, hereafter called *BARD1-V5* WT, and pDEST-FRT/T0-Flag-PALB2, hereafter called *Flag-PALB2* WT, were used. *BARD1-V5* WT was a gift from Masanori Kurihara and Atsushi Iwata [35], and *Flag-PALB2* WT was a gift from Daniel Durocher (Addgene plasmid #71114; http://n2t.net/addgene:71114 (accessed on 2 January 2020); RRID:Addgene_71114) [34]. The corresponding empty vectors (pcDNA6.2-V5 and pDEST FRT/TO-FLAG) were used as controls.

### 2.2. Cell Culture and Transfection

HEK293FT and MDA-MB-231 cells were cultured in DMEM high glucose GlutaMAX™ medium or DMEM medium (Thermo Fisher Scientific, Waltham, MA, USA), respectively, supplemented with 10% FBS (Thermo Fisher Scientific) and 1% PenStrep (Sigma-Aldrich, St. Louis, MO, USA). Both cell lines were maintained in 5% CO_2_ at 37 °C. JetPrime^®^ (Polyplus-Transfection, Illkirch-Graffenstaden, France) was used for transient transfection of the cells according to the manufacturer’s protocol. 

### 2.3. Assessment of BRCA1 Protein Expression by Immunoblotting

For Western blot analyses, cells were lysed in RIPA buffer (supplemented with complete Mini EDTA-free Protease inhibitor cocktail tablets, Roche, Basel, Switzerland) 48 h post transfection, and centrifuged at 13,000× *g* for 10 min at 4 °C. Following measurements of the protein concentration by Pierce BCA protein assay kit (Thermo Fisher Scientific), 5 µg total protein were analysed by SDS-PAGE using 3–5% Tris-Acetate gels (150 V, 75 min) and transferred to a nitrocellulose membrane (30 V, 60 min). One BRCA1 WT sample was always included in each gel to ensure comparable results with the variants investigated. To detect BRCA1 protein, the following antibodies were used: primary anti-BRCA1 (sc-6954, Santa Cruz Biotechnology, Dallas, TX, USA) and secondary m-IgGκ BP-HRP (sc-516102, Santa Cruz). Anti-β-Actin antibody (sc-47778, Santa Cruz) was used as loading control and for quantification of relative BRCA1 protein expression levels. Proteins were visualised using SuperSignal^TM^ West Pico PLUS Chemiluminescent Substrate (Thermo Fisher Scientific) and the ChemiDOC^TM^ MP imaging system. The signals were quantified using the Image Lab^TM^ Software from BioRad (version 6.0). As benign controls for protein expression, the variants p.Lys45Gln, p.Arg504His, and p.Val1378Ile were included (all classified as benign by the ENIGMA expert panel) [36]. As negative controls for protein expression, empty vector and the pathogenic variants p.Ala1708Glu and p.Val1838Gly were included [18,37]. 

### 2.4. RNA Purification and qPCR

HEK293FT cells were seeded in 12-well plates (0.35 × 10^6^ cells/well), and transfected with *BRCA1* WT and variant plasmids. Forty-eight hours after transfection, RNA was purified using RNeasy Mini Kit (QIAGEN) as described by the manufacturer. The quality of the RNA samples was analysed by the Agilent RNA 2200 ScreenTape System. Purified RNA (1 µg) was used to synthesise single-stranded cDNA, applying the SuperScript™ VILO™ cDNA Synthesis Kit (Invitrogen, Waltham, MA, USA). The synthesised cDNA was then used as a template for the analysis of expression of *BRCA1* variants and the housekeeping gene β-actin by qPCR using TaqMan^®^ Gene Expression Assays (Applied Biosystems, Life Technologies). 

### 2.5. MG132 Assay for Assessment of Proteasomal Degradation 

HEK293FT cells were seeded in 12-well plates (0.35 × 10^6^ cells/well) and transfected with *BRCA1* WT and variants. Twenty-four hours post transfection, cells were incubated with 20 µM MG132 (Sigma-Aldrich) dissolved in DMSO or DMSO only for 24 h. Cells were then lysed in 100 µL RIPA buffer (with protease inhibitor). Samples containing 10 µg of protein were analysed by Western blotting, and compared to WT and p.Val1838Gly used as benign and pathogenic controls, respectively. 

### 2.6. Cycloheximide Chase Assay for Measurement of BRCA1 Protein Stability 

HEK293FT cells were seeded in 12-well plates (0.35 × 10^6^ cells/well) and transfected with *BRCA1* WT and variants. Twenty-four hours post transfection, the medium was removed and replaced with fresh medium containing 50 µg/mL cycloheximide (Sigma-Aldrich) dissolved in DMSO or DMSO only. Cells were harvested after 0, 2 and 8 h of treatment. For each time point, the cells were lysed in RIPA buffer (supplemented with protease inhibitor) and frozen at −20 °C immediately after harvest. Centrifugation (13,000 *g*, 10 min, 4 °C) was performed for all samples in parallel >24 h post freezing. Samples containing 5 µg of protein were analysed by Western blotting. As benign controls for protein stability, WT and three benign variants (p.Lys45Gln, p.Arg504His, p.Vall378Ile) were included. As pathogenic controls for protein stability, two variants known to harbour reduced protein stability were used (p.Cys49Tyr and p.Ala1708Glu) [37,38,39]. The resulting % protein expressions presented are relative to the protein levels for the corresponding variant at the starting point (0 h, corresponding to 100%).

### 2.7. Fractionation Assay for Assessment of Subcellular Localisation 

Subcellular localisation was tested by a fractionation assay separating the cytosolic and nuclear cell fractions [40,41]. HEK293FT cells seeded in 10 cm dishes (4.8 × 10^6^ cells/dish) were transfected with 10 µg plasmid encoding *BRCA1* WT or variants. Forty-eight hours post transfection, the cells were washed in PBS and pelleted at 1200 rpm for 5 min, before resuspending the cells in 250 µL buffer A (10 µM HEPES pH 7.8, 1.5 mM MgCl_2_, 10 mM KCl, 0.10% IGEPAL, 0.5 mM DTT, EDTA free protease inhibitor) and incubating for 30 min. The suspension was then pelleted at 13,000 rpm for 5 min at 4 °C. The resulting supernatant, which is the cytosolic fraction, was then frozen at −80 °C for later analyses. The pellet was washed once with 100 µL buffer A and resuspended in 100 µL buffer B (20 mM HEPES pH 7.8, 420 mM NaCl, 1.5 mM MgCl_2_, 0.2 mM EDTA, 0.5 mM DTT, EDTA free protease inhibitor) by pipetting up/down 30 times. After 30 min incubation on ice with vortexing every minute, the resuspension was centrifuged at 13,000 rpm for 15 min at 4 °C. The resulting supernatant, which is the nuclear fraction, was then frozen at −80 °C for later analyses. Cytosolic and nuclear samples containing 5 µg of total protein were analysed by Western blotting. Anti-Topoisomerase IIα (D10G9, Cell Signaling Technology, Danvers, MA, USA) and Anti-HSP 90α/β (sc-13119, Santa Cruz) were used to confirm the purity of the nuclear and cytosol fractions, respectively, and were used for normalisation. The % of BRCA1 protein in each fraction was then calculated, and the level of protein in the nucleus was presented. 

### 2.8. Co-Immunoprecipitation Assay 

HEK293FT cells were seeded in 10 cm Petri dishes (4.8 × 10^6^), and co-transfected with 5 µg plasmid encoding *BRCA1* WT or the selected *BRCA1* variants in combination with either 5 µg plasmid encoding *BARD1-V5* WT or *Flag*-*PALB2* WT. After 48 h, cells were lysed in 500 µL ice-cold IP Lysis/Wash Buffer (supplemented with protease inhibitor) per dish. The cell lysate was centrifuged at 13,000× *g* for 10 min at 4 °C, before measuring protein concentration by Pierce BCA protein assay kit. Co-immunoprecipitation (Co-IP) was performed using the Dynabeads^TM^ Protein G Immunoprecipitation Kit (Invitrogen) according to the manufacturer’s protocol, with the following specifications: 5 µg of V5 antibody (for WT *BARD1*-*V5*) or 5 µg of Flag antibody (for WT *Flag*-*PALB2*) was coupled to 50 µL magnetic beads. Equal amounts of cell lysate proteins (2 mg, input) were incubated with the antibody-coupled beads for 90 min at 4 °C. After non-denaturing elution of the protein complexes, the proteins bound to the beads (IP) were separated by SDS-PAGE, and BRCA1 WT or variants in combination with BARD1-V5 or Flag-PALB2 were visualised by Western blotting using anti-V5 (46-0705, Invitrogen) or anti-Flag (F1804, Sigma-Aldrich), respectively. BRCA1 protein levels in the IP samples were quantified and normalised to the anti-V5 signal or anti-Flag signal in the IP samples. The data for each of the variants were presented as % compared to WT (set to 100%). As controls for the Co-IP assay with BRCA1-PALB2, the benign variant p.Val1378Ile and the pathogenic variant p.Met1411Thr (both located in the coiled-coil domain of BRCA1), were included [42,43]. As controls for the Co-IP assay with BRCA1-BARD1, the benign variant p.Lys45Gln and the pathogenic variant p.Cys39Tyr (both located in the RING domain of BRCA1) were included [38,39]. 

### 2.9. Statistics 

All experiments were carried out on at least three independent occasions unless otherwise specified in the figure legends, with the exception of the empty vector, which was performed in one replicate only. The standard deviations were calculated for WT and each variant in all assays. The statistical significance was evaluated with the Student’s *t*-test with *p* values < 0.05. 

### 2.10. Assessment of Variant Classifications

The Alamut Software (Version 2.15, SOPHiA GENETICS) and the Human Gene Mutation Database (HGMD) professional 2022.1 (QIAGEN) were used for gathering information on the *BRCA1* variants. Reinterpretation of the variants was performed based on new knowledge using the ACMG-AMP criteria supplemented with the *BRCA1/BRCA2* gene-specific criteria by CanVIG-UK [9,16].

## 3. Results

### 3.1. Effects of BRCA1 Variants on Protein Expression

**Figure 2:**
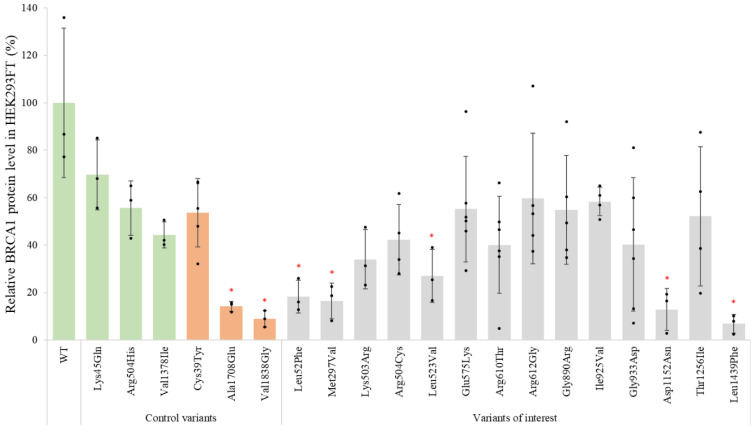
Protein expression levels of BRCA1 variants determined by Western blot analysis: HEK293FT cells were transiently transfected with *BRCA1* WT, known benign and pathogenic control variants and 14 missense *BRCA1* VUSs. The black dots represent individual normalised band intensities. Each column represents the mean of three to six independent replicates (*n* = 3–6). The benign (green) and pathogenic (orange) control variants are grouped to the left. Variants marked with a red * indicate *p* < 0.05. Error bars represent standard deviation.

To test the effect of the selected *BRCA1* missense variants (Figure 1) on the protein expression level, the corresponding plasmids were transfected into HEK293FT cells and the cell lysates were analysed by Western blot analysis (Supplementary Figure S1A). As expected, a band located just above 220 kDa, corresponding to the theoretical molecular weight of mCherry-BRCA1 (248 kDa), was detected for both the WT and variants. Nine variants had similar relative expression levels as the WT (100%) and/or benign controls (44–70%). Four of the variants (p.Leu52Phe, p.Met297Val, p.Asp1152Asn and p.Leu1439Phe) displayed severely reduced protein levels, i.e., <20% protein compared to WT, similar to the included pathogenic controls (9–14%) (Figure 2). In addition, the variant p.Leu523Val was found to have reduced protein expression (27%) compared to the WT, at an intermediate expression level between pathogenic (9–14%) and benign controls (44–70%). For comparison, the assay was repeated in MDA-MB-231 cells, where a similar trend for protein expression was seen (Supplementary Figure S1B)

### 3.2. qPCR for Assessment of mRNA Levels 

**Figure 3:**
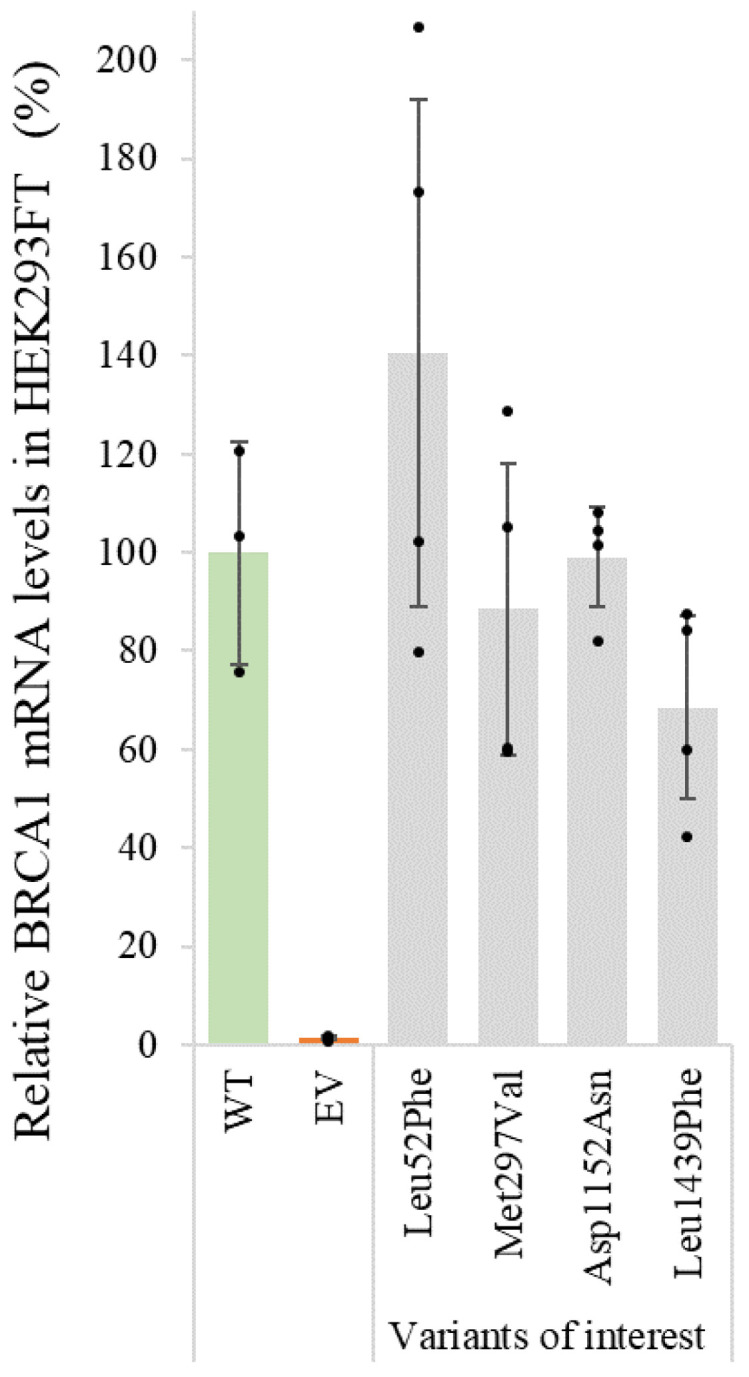
mRNA levels of *BRCA1* variants in HEK293FT cells determined by qPCR: HEK293FT cells were transfected with plasmids encoding *BRCA1* WT and the four variants found to be expressed at protein levels lower or similar to the included pathogenic controls, as shown in Figure 2. Each column represents the mean of three or four independent replicates (*n* = 3–4), and the black dots represent individual values after normalisation using actin. Error bars represent standard deviation.

The four protein variants (p.Leu52Phe, p.Met297Val, p.Asp1152Asn and p.Leu1439Phe) found to be expressed at levels lower or similar to the included pathogenic controls in HEK293FT cells were subsequently analysed by qPCR to investigate if the low protein expression was caused by a reduction of the mRNA levels. After normalisation of the data by actin, the relative mRNA levels for each variant compared to the *BRCA1* WT were calculated (Figure 3). The results suggest that the plasmids encoding p.Leu52Phe, p.Met297Val, p.Asp1152Asn and p.Leu1439Phe produce similar amounts of mRNA as the WT plasmid. Thus, for these variants, reduced protein levels are unlikely to be caused by reduced transcription or transfection efficiency, but are more likely caused by increased protein degradation or reduced stability. 

### 3.3. MG132 Assay for Assessment of Proteasomal Degradation 

**Figure 4:**
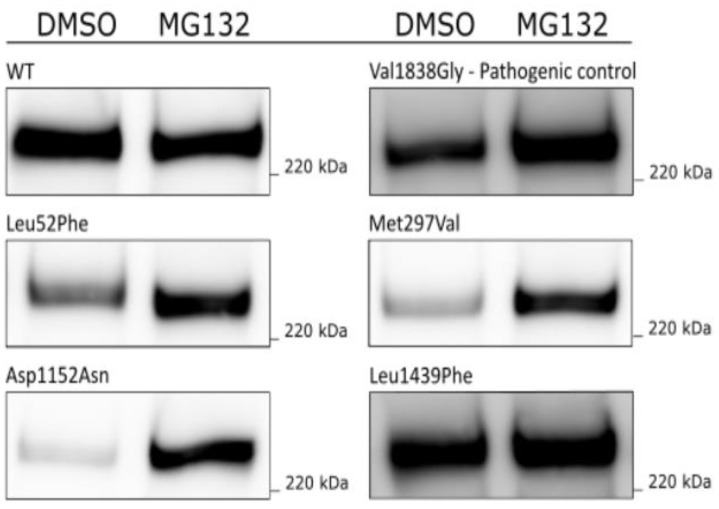
Assessment of proteasomal degradation of BRCA1 variants by treatment with MG132: HEK293FT cells were transiently transfected with *BRCA1* WT, the pathogenic control p.Val1838Gly, and four missense *BRCA1* VUSs. Cells were treated with 20 µM MG132 or DMSO for 8 h after transfection. BRCA1 (220 kDa) was detected with anti-BRCA1 antibody.

To check whether the low protein levels in HEK293FT cells observed for p.Leu52Phe, p.Met297Val, p.Asp1152Asn and p.Leu1439Phe could be due to degradation by the ubiquitin–proteasome system, transfected HEK293FT cells were treated with proteasome inhibitor MG132 for 24 h. As shown in Figure 4, protein expression clearly increased for the pathogenic control (p.Val1838Gly) and three of the variants (p.Leu52Phe, p.Met297Val and p.Asp1152Asn) after treatment with MG132, compared to the control samples treated with DMSO only. For the variant p.Leu1439Phe, comparable amounts of protein were observed in the MG132 treated sample and the DMSO control sample. 

### 3.4. Cycloheximide Chase Assay for Assessment of Protein Stability 

**Figure 5:**
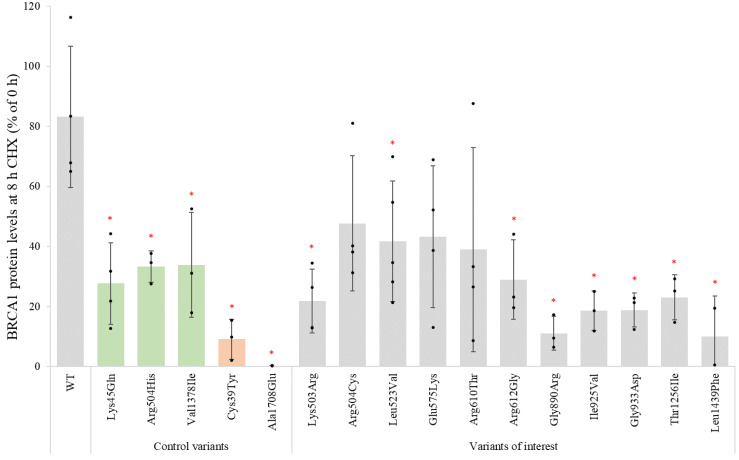
Assessment of BRCA1 protein variant stability after 8 h by cycloheximide chase assay: HEK293FT cells were transiently transfected with *BRCA1* WT, known benign and pathogenic control variants and 11 missense *BRCA1* VUSs. The columns show normalised mean protein levels of three to five independent replicates (*n* = 3–5) after 8 h of treatment with cycloheximide relative to the levels at 0 h of treatment (100%) for each individual variant. The black dots represent individual normalised band intensities. Error bars represent standard deviation. The benign and pathogenic control variants are coloured green and orange, respectively. Variants marked with a red * indicate a significant reduction in protein stability compared with WT protein (*p* < 0.05).

For the BRCA1 variants which showed protein expression levels above 20% compared to the BRCA1 WT protein in the Western blot analysis (Figure 2), including the variant p.Leu1439Phe, which showed equal amounts of protein in the MG132 assay (Figure 4), the protein stability was analysed by cycloheximide chase assay to follow protein degradation over time in transfected HEK293FT cells. The results from one representative replicate after 0, 2 and 8 h of treatment with the protein synthesis inhibitor cycloheximide, compared to DMSO only for a minor selection of variants (p.Gly890Arg and p.Leu1439Phe) and controls (p.Arg504His and p.Ala1708Glu), are shown in Supplementary Figure S2. Figure 5 shows the mean percentage BRCA1 protein level remaining for each variant in transfected HEK293FT cells treated with cycloheximide for 8 h. For the BRCA1 WT, the protein expression level decreased to 83% after 8 h treatment with cycloheximide. For all the variants, including the benign controls, a more prominent degradation of BRCA1 protein was observed during cycloheximide treatment. The protein levels for all the benign controls were reduced to 28–34%, while there was, respectively, 0% and 9% protein detected for the pathogenic controls p.Ala1708Glu and p.Cys39Tyr. Protein levels of the two BRCA1 VUSs, p.Gly890Arg and p.Leu1439Phe, were reduced to 11% and 10%, respectively, after 8 h of cycloheximide treatment, similar to the pathogenic controls. The nine remaining variants showed a reduction in protein levels comparable to the benign controls after 8 h of treatment. 

### 3.5. Assessment of Subcellular Localisation by Fractionation Assay 

According to the literature, the BRCA1 protein is known to be mainly located in the nucleus, and the two NLS of BRCA1 are located at aa 503–508 and 607–614 [44]. To investigate if some of the variants of interest could alter the nuclear localisation of the protein, subcellular localisation was assessed by a nuclear/cytosol fractionation assay. This was especially interesting to evaluate for the variants p.Lys503Arg, p.Arg504Cys, p.Arg610Thr and p.Arg612Gly, which are all located within the nuclear localisation sequences. Similar to the BRCA1 WT protein (84% located in the nucleus), all analysed variants were found to be mainly located in the nucleus fraction (Supplementary Figure S3). 

### 3.6. Assessment of Protein–Protein Interactions with BARD1 and PALB2 by Co-Immunoprecipitation 

**Figure 6:**
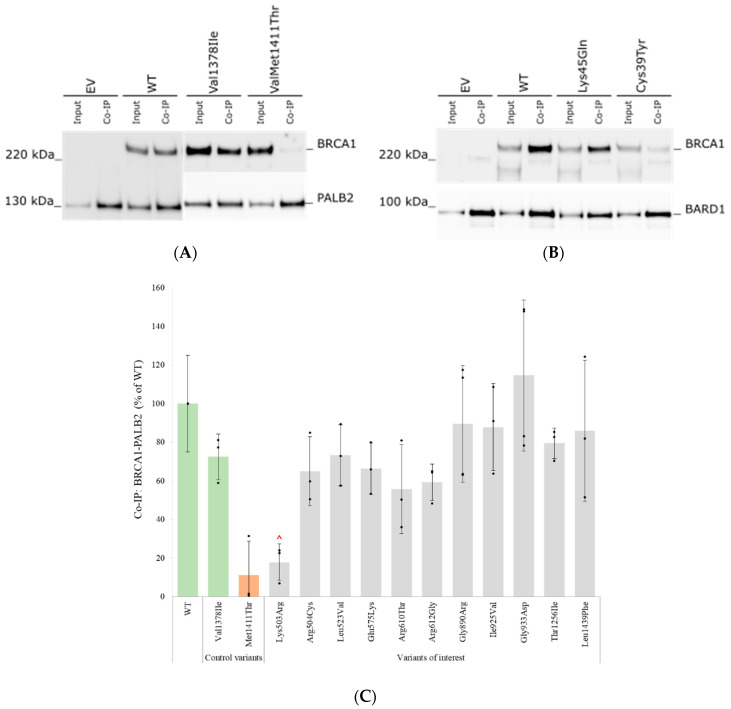

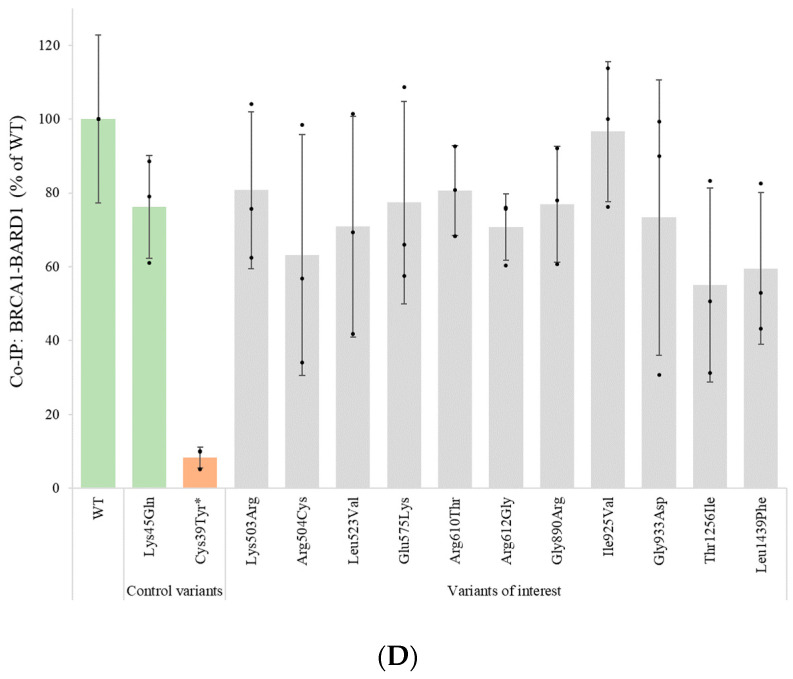
Assessment of protein interactions between BRCA1 and BARD1 or PALB2 by Co-IP assay: (**A**) HEK293FT cells were transiently co-transfected with EV or *BRCA1* construct together with *Flag-PALB2*. Cells were harvested 48 h post transfection and co-immunoprecipitation (Co-IP) was performed. Input = input cell lysates, Co-IP = eluates from the Flag-column. BRCA1 (220 kDa) was detected with anti-BRCA1. PALB2-Flag (130 kDa) was detected with anti-Flag. Representative results from one of in total three experiments are shown. (**B**) Identical experiment to (**A**), with *BARD1*-V5 and V5 antibody coupled to the magnetic beads. BARD1-V5 (100 kDa) was detected with anti-V5. (**C**) Quantified results from BRCA1-PALB2 Co-IP. Western blot bands from three biological replicates were quantified by Image Lab software (n = 3). Black dots represent individual normalised band intensities. Graphs represent mean % compared to the WT. Error bars represent standard deviation. The benign (green) and pathogenic (orange) control variants are grouped to the left. In the initial analysis, the variant p.Lys503Arg appeared to have a reduced binding to PALB2, but this interaction was shown to be similar to the WT/benign controls when quantifying against the amount of the variant input samplemarked by a red ∧). (**D**) Identical experiment to (**C**), but with BRCA1-BARD1 Co-IP.

Co-IP assays were performed to test the potential effect of the VUSs on the binding of the BRCA1 protein to two of its binding partners: BARD1 and PALB2. The resulting Western blots for the WT, EV, control variants and a selection of the analysed VUSs are illustrated in Figure 6A,B. As seen in the blots, the BRCA1 WT protein captured both PALB2 (Figure 6A) and BARD1 (Figure 6B). A strong binding to the respective benign controls p.Val1378Ile and p.Lys45Gln was observed, and a weak binding to the respective pathogenic controls p.Met1411Thr and p.Cys39Tyr. Mean values for all variants (% binding capacity compared to WT) are shown in Figure 6C,D. In the initial analysis, the variant p.Lys503Arg appeared to have a reduced binding to PALB2 (Figure 6C), but this interaction was shown to be similar to the WT/benign controls when quantifying against the amount of the variant input sample. Thus, none of the variants of interest were showed to have significantly reduced binding to either BARD1 or PALB2.

### 3.7. Summary of Functional Assays

When summarising the data from each of the different functional assays (Table 1), five *BRCA1* variants were found to have one or more features strongly deviating from the WT protein, while the remaining nine variants showed no or only minor deviations. The data gathered throughout the study were combined with other relevant information and used to suggest an updated classification of the pathogenicity of these rare *BRCA1* variants (Table 2). The molecular properties and previously known information on each of these variants will be discussed below.

## 4. Discussion 

In this study, we have examined the effects of 14 rare *BRCA1* missense VUSs to investigate the hypothesis stating that no pathogenic *BRCA1* missense variants are present outside of known protein domains in BRCA1 [24,25,26]. We have investigated the effect of each variant by multiple functional protein assays, using the full-length BRCA1 protein to better mimic the native state of the protein.

### 4.1. BRCA1 Protein Domains and Amino Acid Conservation 

Use of the BP1 criteria in the gene-specific *BRCA1/BRCA2* guidelines from CanVIG-UK, indicating that no pathogenic *BRCA1* missense variants are present outside of known protein domains, was debated in our “*BRCA1* Norway” publication [16,33]. Counterarguments stated that amino acid residues located outside well-established domains in the primary structure of the polypeptide chain can potentially interact with or become part of important structural and functional elements in the native folded three-dimensional structure of the BRCA1 protein. Thus, the replacement of amino acid residues located outside an important domain in the primary structure could possibly affect both structure and function of the protein. In fact, it has been suggested that the majority of the loss of function missense mutation is indirectly caused by the destabilisation of the protein’s three-dimensional-structure, rather than directly disrupting important functional characteristics such as binding sites [37,49,50,51]. In contrast to the highly conserved RING and BRCT domains, for which the structure is known, it has been suggested that the central 1500 residue region of BRCA1 acts as a long flexible scaffold for intermolecular interactions, even though the central region lacks substantial conserved motifs [7]. Such intrinsically non-conserved disordered regions are known to obtain a more folded structure upon interaction with its protein partners [7,29,30,31,32]. Thus, although a disordered region in the absence of its binding partners, this central region might still be functionally important in the DNA damage response [7]. 

### 4.2. Protein Expression and Protein Stability of the BRCA1 Variants 

For many genes, the protein expression level of a variant is known to correlate with the pathogenicity of the variant [40,41]. In contrast, it has been demonstrated that BRCA1 protein variants displaying low protein levels may still sustain structure/function similar to the WT protein, and that variants with protein levels similar to the WT protein may fail to sustain function [13,52]. These studies were, however, performed by expression of isolated protein domains and not the full-length BRCA1 protein. The effect of missense changes on the expression of full-length BRCA1 protein, particularly those located outside of the known domains, has to our knowledge not previously been thoroughly investigated. We, therefore, aimed to investigate the effect of our selected *BRCA1* variants on protein expression levels in HEK293FT cells by Western blotting. The benign control variants showed reduced protein level (44–70%) compared to the WT protein, but considerably higher levels than the pathogenic control variants (9–14%). In concordance with previous studies [52], this indicates that even significantly reduced BRCA1 expression levels are sufficient to maintain BRCA1 protein functions, and that protein expression levels do not necessarily correlate with the level of protein activity. In addition, the lower threshold for BRCA1 protein expression associated with pathogenicity is currently unknown. Nine of the investigated variants showed protein expression levels comparable to the benign controls/WT protein (27–60%), while the four variants (p.Leu52Phe, p.Met297Val, p.Asp1152Asn and p.Leu1439Phe) showed reduced protein levels in the range of the pathogenic controls (7–18%). Low protein expression can be caused by, among others, low transcription levels, protein instability or increased protein degradation. Although protein expression analyses alone are not adequate to distinguish between benign and pathogenic variants, protein expression analysis can, in combination with additional protein assays, still provide important insights regarding the underlying mechanism for the loss of protein function. To investigate the cause of the reduced protein levels, therefore, we analysed the four variants, p.Leu52Phe, p.Met297Val, p.Asp1152Asn and p.Leu1439Phe, using qPCR. The mRNA levels for the four variants were found to be in the same range as the WT, indicating that the underlying mechanism for the low protein expression levels is at the protein level. 

It has previously been shown that several missense variants in the BRCT domain lead to increased susceptibility to degradation of BRCA1 and destabilisation of the protein structure, by, among others, the ubiquitin–proteasome system [53,54,55,56,57,58]. When investigating the aforementioned four variants by inhibiting the ubiquitin–proteasome degradation pathway by MG132, an increased protein level was observed for three of the variants (p.Leu52Phe, p.Met297Val and p.Asp1152Asn) and the pathogenic control p.Val1838Gly, which has been previously shown to have reduced protein levels in HEK293 cells [18]. This indicates that these variants, of which two are located outside of known protein domains, make the BRCA1 protein more prone to ubiquitin-mediated degradation. In contrast, the variant p.Leu1439Phe seems not to be removed by the proteasomal system. 

To evaluate the protein stability of the BRCA1 variants over time, a cycloheximide chase assay was performed for the BRCA1 variants showing protein expression levels comparable to the benign controls/WT protein. In addition, the p.Leu1439Phe variant, which was found not to be removed by the proteasomal system, was included. The BRCA1 WT protein showed a stability of 83% after treatment with cycloheximide. All benign control variants, surprisingly, illustrated 28–34% protein levels compared to the WT protein after eight hours, indicating that a protein variant could have pronounced reduction in stability without affecting the pathogenicity. In comparison, the pathogenic control variants p.Ala1708Glu and p.Cys39Tyr showed, respectively, 0% and 9% protein expression after treatment with cycloheximide. Similar to the two pathogenic controls, the two VUSs, p.Gly890Arg and p.Leu1439Phe, showed severely reduced protein stability compared to the WT protein (11% and 10%, respectively). After cycloheximide treatment, four of the VUSs (p.Lys503Arg, p.Ile925Val, p.Gly933Asp and p.Thr1256Ile) demonstrated protein levels in the range 19–23% of the WT, at an intermediate level between the pathogenic and benign controls. The remaining five VUSs showed protein levels comparable to the benign controls (29–48%). In order to improve the capacity of the assay to better discriminate the benign/pathogenic thresholds, more pathogenic and benign controls should be included in this assay [10]. 

### 4.3. The Effect of BRCA1 Variants on BARD1 and PALB2 Interaction 

The BRCA1 protein is known to interact with a myriad of other proteins. Among others, BRCA1 interacts with BARD1 though the RING domain, and with PALB2 through the coiled-coil domain. Although only one of the 14 VUSs analysed in this study is located in the RING domain, and none in the coiled-coil domain, we wanted to investigate if any of our variants of interest could alter these interactions. The effect of an abolished BRCA1–BARD1 interaction was illustrated by the pathogenic BRCA1 control variant p.Cys39Tyr, located in the RING domain. Even though initial protein levels of the variant appeared to be within the normal range (Figure 2, this variant demonstrated reduced binding to BARD1 during Co-IP (Figure 6D) and severely reduced protein stability in the cycloheximide chase assay (Figure 5). The reduced stability can potentially be explained by the fact that variants impairing the interaction between BRCA1 and BARD1 can result in the proteolytic degradation of both proteins and, thus, our results are in agreement with previously published data [38,39]. In contrast, the benign control p.Lys45Gln, which is also located within the RING domain, showed normal protein expression levels, stability and BRCA1-BARD1 binding. In the BRCA1-PALB2 assay, the variant p.Met1411Thr located in the coiled-coil domain was used as a pathogenic control. This missense variant has, in agreement with our results, previously been shown to abolish BRCA1 interaction with PALB2 [42,43]. The benign control p.Val1378Ile, equally located in the coiled-coil domain, showed normal BRCA1-PALB2 binding. However, none of the variants of interest showed significantly reduced binding to either BARD1 or PALB2. 

### 4.4. Variant Interpretation of the Investigated VUSs

Even though the general protein-based analyses performed in this study are not among the functional assays suggested by CanVIG-UK, our data indicate that the new knowledge could provide useful information regarding the pathogenicity of variants located outside of the known protein domains of BRCA1. We, therefore, wanted to investigate whether our newly achieved functional data could contribute to the re-classification of the 14 investigated VUSs (Table 2). In our study, the three variants p.Leu52Phe, p.Met297Val and p.Asp1152Asn were shown to have reduced protein expression levels (<20% protein compared to WT), probably due to removal by proteasomal degradation. The p.Leu52Phe variant has previously been functionally assessed by others, with conflicting results. This variant has been shown to have normal binding to BARD1 [59,60], normal HRR activity [59,61], and normal saturation genome editing assay [11]. However, defective ubiquitination [19], impact on centrosome duplication [62], and changes in E3 ligase activity [60] have also been reported. Furthermore, the variant allele frequency in the East Asian population is 0.09% according to the gnomAD database, which is above the expected frequency of a pathogenic variant (BS1 criteria) [16]. In all cases where there were conflicts between our newly achieved functional data and the data in any of the five functional BRCA1 protein studies suggested by the CanVIG-UK, we chose not to include the functional evidence criteria (BS3 or PS3) when classifying the variants [11,12,13,14,15,16]. Due to the conflicting evidence from functional studies, we, therefore, still classify p.Leu52Phe as a VUS. For the p.Met297Val variant, no previous experimental evidence demonstrating its impact on protein function has been reported. In cases where the variants of interest were not investigated in any of the functional studies recommended by CanVIG-UK, we chose to apply the functional criteria (PS3 or BS3) as supportive strength. Thus, due to a lack of evidence, p.Met297Val is also still assessed as a VUS. The variant p.Asp1152Asn is predicted as benign by in silico tools, and according to CanVIG-UK this variant could, therefore, theoretically be classified as likely benign (BP1 and BP4 criteria). This variant has been shown to harbour normal HRR activity [12,62], and to be neutral in cisplatin and olaparib assays [12], which would qualify for the BS3 criteria. However, in our study, the variant showed low protein expression when analysed in HEK293FT cells (Figure 2), and even lower in MDA-MB-231 cells (Supplementary Figure S1B). Due to conflicting functional evidence, we therefore still chose to classify p.Asp1152Asn as a VUS due to the remarkably low protein expression levels and increased proteasomal degradation, and suggest that the variant should be analysed by further studies. In the initial Western blot analysis, the variant p.Gly890Arg showed similar protein expression levels as the benign controls, but a severely reduced protein stability over time compared to the WT in the cycloheximide chase assay. The variant p.Leu1439Phe was shown to have both reduced initial protein expression levels and reduced protein stability over time. Both p.Gly890 and p.Leu1439 are weakly conserved amino acids. For p.Gly890Arg, no experimental evidence demonstrating its impact on protein function has previously been reported. The variant p.Leu1439Phe has been found to be neutral in HRR and a cisplatin sensitivity assay, but showed inconclusive results in an olaparib sensitivity assay. Due to our findings of reduced protein stability, we still assess these variants as VUSs. 

For the nine remaining variants, no significant effect on the BRCA1 protein expression, protein stability, subcellular localisation or BARD1/PALB2 interaction was observed. When including information on allele frequency, conservation, the literature, and in silico predictions, seven of these variants were suggested to be reclassified as likely benign. The original and new classifications for each variant are summarised in Table 2. 

## 5. Conclusions

In this study, we have assessed the effect of 14 *BRCA1* missense VUSs using the full-length protein and multiple functional assays, aiming to investigate the hypothesis stating that no pathogenic *BRCA1* missense variants are present outside of protein domains with known function. Although our findings should be confirmed using additional pathogenic and benign control variants to improve the discrimination, the findings indicate that variants located outside the RING, BRCT and coiled-coiled domains could also affect the BRCA1 protein, and that the BP1 criteria should be used with care. This study also illustrates the importance of not relying on one functional assay only, but rather including several assays when investigating variants in the multifunctional BRCA1 protein.

## Figures and Tables

**Table 1:** Summary of results from functional assays.

Variant cDNA	Protein	Protein Expression (% of WT)	qPCR (% of WT)	% Reduction in Protein Levels (after 8 h CHX Treatment)	Inhibition of Proteolytic Degradation (MG132)	Co-IP BARD1 (% of WT)	Co-IP PALB2 (% of WT)	Nuclear Localization (% of Total)	Summary Functional Assays
	WT protein	100 ± 31	100 ± 23	83 ± 23	Equal amounts	100 ± 23	100 ± 25	84 ± 10	
c.154C > T	p.Leu52Phe	18 ± 7	141 ± 51		Increased protein levels			77 ± 5	Reduced protein expression
c.889A > G	p.Met297Val	16 ± 8	89 ± 30		Increased protein levels			82 ± 7	Reduced protein expression
c.1508A > G	p.Lys503Arg	34 ± 12		22 ± 11		81 ± 21	18 ± 9	81 ± 12	No deviations detected
c.1510C > T	p.Arg504Cys	42 ± 15		48 ± 23		63 ± 33	65 ± 18	71 ± 16	No deviations detected
c.1567T > G	p.Leu523Val	27 ± 11		42 ± 20		71 ± 30	73 ± 16	88 ± 1	No deviations detected
c.1723G > A	p.Glu575Lys	55 ± 22		43 ± 24		77 ± 27	66 ± 13	87 ± 10	No deviations detected
c.1829G > C	p.Arg610Thr	40 ± 20		39 ± 34		81 ± 12	56 ± 23	87 ± 2	No deviations detected
c.1834A > G	p.Arg612Gly	60 ± 28		29 ± 13		71 ± 9	59 ± 9	88 ± 9	No deviations detected
c.2668G > A	p.Gly890Arg	55 ± 23		11 ± 6		77 ± 16	89 ± 30	90 ± 10	Reduced protein stability
c.2773A > G	p.Ile925Val	58 ± 6		19 ± 7		97 ± 19	88 ± 23	95 ± 6	No deviations detected
c.2798G > A	p.Gly933Asp	40 ± 28		19 ± 6		73 ± 37	115 ± 39	88 ± 12	No deviations detected
c.3454G > A	p.Asp1152Asn	13 ± 9	99 ± 10		Increased protein levels			88 ± 10	Reduced protein expression
c.3767C > T	p.Thr1256Ile	52 ± 29		23 ± 7		55 ± 26	79 ± 8	90 ± 2	No deviations detected
c.4315C > T	p.Leu1439Phe	7 ± 4	69 ± 19	10 ± 13	Equal amounts	60 ± 20	86 ± 37	82 ± 13	Reduced protein expression and stability
Benign control variants									
c.133A > C	p.Lys45Gln	70 ± 15		28 ± 14		76 ± 14			
c.1511G > A	p.Arg504His	56 ± 11		33 ± 5					
c.4132G > A	p.Val1378Ile	44 ± 5		34 ± 17			72 ± 11		
Pathogenic control variants									
c.116G > A	p.Cys39Tyr			9 ± 7		8 ± 3			
c.4232T > C	p.Met1411Thr						11 ± 18		
c.5513T > G	p.Val1838Gly	9 ± 3			Increased protein levels				
c.5123C > A	p.Ala1708Glu	14 ± 2		0 ± 0					

Abbreviations: CHX, cycloheximide; WT, wild type.

**Table 2:** Characteristics and resulting reclassification for the studied *BRCA1* variants.

Variant	Region/Domain	Functional Studies Recommended by CanVIG-UK *	Results in Functional Assays in This Study	GnomAD MAF % (Allele Count) **	REVEL ***	Splicing ****	ClinVar Classifications	CanVIG-UK Criteria	Original Class *****	Revised Class
c.154C > T p.(Leu52Phe)	RING	Functional HRR [15], functional saturating genome assay [11]	Reduced protein expression and increased proteasomal degradation	0.09354 (24)	0.68	Possible effect	VUSx7, LBx4, Bx2	BS1_strong	VUS	VUS
c.889A > G p.(Met297Val)			Reduced protein expression and increased proteasomal degradation	- (1)	0.58		VUSx6	BP1, PS3_sup, PM2_sup	LB, VUS	VUS
c.1508A > G p.(Lys503Arg)	NLS		No deviations detected	0.0003240 (2)	0.76		VUSx4, LBx3	BP1, BS3_sup, PP3, PM2_sup	LB, VUS	VUS
c.1510C > T p.(Arg504Cys)	NLS		No deviations detected	0.0003240 (4)	0.65		VUSx10	BP1, BS3_sup, PM2_sup	VUS	LB
c.1567T > G p.(Leu523Val)			No deviations detected	- (1)	0.61		VUSx2	BP1, BS3_sup, PM2_sup	VUS	LB
c.1723G > A p.(Glu575Lys)			No deviations detected	0.0009700 (5)	0.62		VUSx6	BP1, BS3_sup, PM2_sup	VUS	LB
c.1829G > C p.(Arg610Thr)	NLS		No deviations detected	- (0)	0.74		VUSx1	BP1, BS3_sup, PP3, PM2_mod	VUS	VUS
c.1834A > G p.(Arg612Gly)	NLS		No deviations detected	- (0)	0.60		VUSx6, LBx1, Bx1 (ENIGMA)	BS3_sup, BP6, PM2_mod	VUS	LB
c.2668G > A p.(Gly890Arg)			Reduced protein stability	- (0)	0.39	Possible effect	VUSx3, LBx5	PS3_sup, PM2_mod	VUS	VUS
c.2773A > G p.(Ile925Val)			No deviations detected	0.0003240 (2)	0.19		VUSx4, LBx3	BP1, BP4, BS3_sup, PM2_sup	LB, VUS	LB
c.2798G > A p.(Gly933Asp)		Neutral in cisplatin, olaparib and DR-GFP HRR assays [12]	No deviations detected	- (0)	0.29	Possible effect	VUSx5, LBx1	BS3_strong, BS4_sup, PM2_mod	LB, VUS	LB
c.3454G > A p.(Asp1152Asn)		Neutral in cisplatin, olaparib and DR-GFP HRR assays [12]	Reduced protein expression and increased proteasomal degradation	0.004516 (10)	0.37		VUSx10, LBx6, Bx1	BP1, BP4	LB	VUS •
c.3767C > T p.(Thr1256Ile)			No deviations detected	- (0)	0.42		VUSx1	BP1, BS3_sup, PM2_mod	VUS	LB
c.4315C > T p.(Leu1439Phe)		Neutral in cisplatin and DR-GFP HRR assays, not clear in olaparib assay [12]	Reduced protein expression and stability	0.0007760(3)	0.25		VUSx3, LBx1	BP1, BP4, PM2_sup	LB, VUS	VUS •

* According to the CanVIG-UK *BRCA1* specific guideline for variant interpretation, five functional protein studies are suggested with specific recommendations regarding the strength of their respective functional evidence [11,12,13,14,15,16]. ** Minor allele frequencies were retrieved from GnomAD (v2.1.1., non-cancer) Popmax Filtering AF (95% confidence) [45]. As recommended by Garrett et al., the PM2 evidence was ignored when determining the final variant classifications in the presence of evidence towards benignity [46]. *** REVEL was used to assess in silico predictions with a benign cut off at or below 0.4 and a pathogenic cut off at or above 0.7 as recommended in the Best Practice Guidelines for Variant Classification by CanVIG-UK [47,48]. **** When investigating the variants’ effect on splicing, SpliceSiteFinder-like and MaxEntScan in Alamut were used as recommended by CanVig-UK [16]. ***** Original classification in the “*BRCA1* Norway” study [33]. • These variants could theoretically be classified as likely benign (BP1 and BP4 criteria) according to CanVIG-UK [16,47], but due to conflicting functional evidence they were still classified as VUSs. Abbreviations: B, Benign; LB, Likely Benign; MAF, Minor Allele Frequency; VUS, Variant of Uncertain Significance.

## Data Availability

The data generated during the current study are available from the corresponding author upon request.

## Supplementary Materials

The following supporting information can be downloaded at: https://www.mdpi.com/article/10.3390/genes14020262/s1, Table S1: BRCA1 variants included in this study; Figure S1—Protein expression levels of BRCA1 variants determined by western blot analysis; Figure S2—Assessment of protein stability after 0, 2 and 8 hours treatment with cycloheximide or DMSO only; Figure S3—Assessment of nuclear localisation of BRCA1 variants by cellular fractionation assay.
