# Supplementary material for: Functional Analyses of Rare Germline Missense BRCA1 Variants Located within and outside Protein Domains with Known Functions
Source: Genes (Basel). 2023 Jan 19;14(2):262. doi: 10.3390/genes14020262 (PMC9957003; doi:10.3390/genes14020262)
Supplement: Supplementary file 1 [file genes-14-00262-s001.zip › genes-2129374-supplementary.pdf]

Supplementary table

Supplementary Table S1 – *BRCA1* variants included in this study.

**Variant**<br>**cDNA**	**Protein**
**Variants of uncertain significance**	
c.154C>T	p.Leu52Phe
c.889A>G	p.Met297Val
c.1508A>G	p.Lys503Arg
c.1510C>T	p.Arg504Cys
c.1567T>G	p.Leu523Val
c.1723G>A	p.Glu575Lys
c.1829G>C	p.Arg610Thr
c.1834A>G	p.Arg612Gly
c.2668G>A	p.Gly890Arg
c.2773A>G	p.Ile925Val
c.2798G>A	p.Gly933Asp
c.3454G>A	p.Asp1152Asn
c.3767C>T	p.Thr1256Ile
c.4315C>T	p.Leu1439Phe
**Benign control variants**	
c.133A>C	p.Lys45Gln
c.1511G>A	p.Arg504His
c.4132G>A	p.Val1378Ile
**Pathogenic control variants**	
c.116G>A	p.Cys39Tyr
c.4232T>C	p.Met1411Thr
c.5513T>G	p.Val1838Gly
c.5123C>A	p.Ala1708Glu

Supplementary figures

A

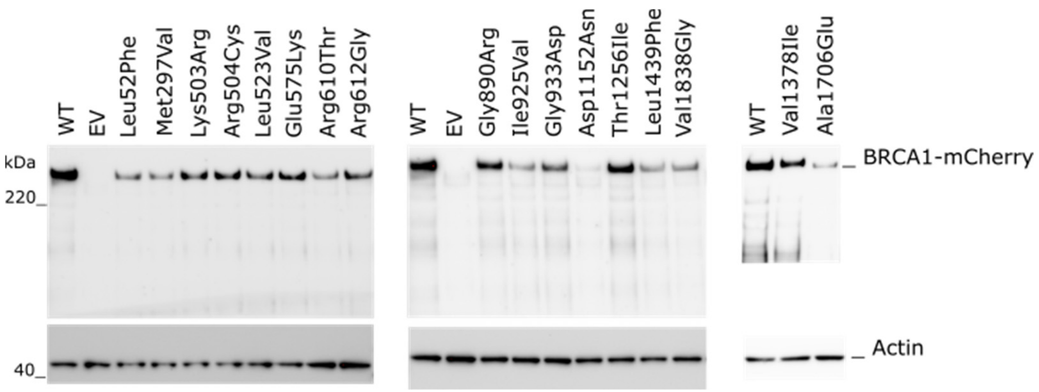

B

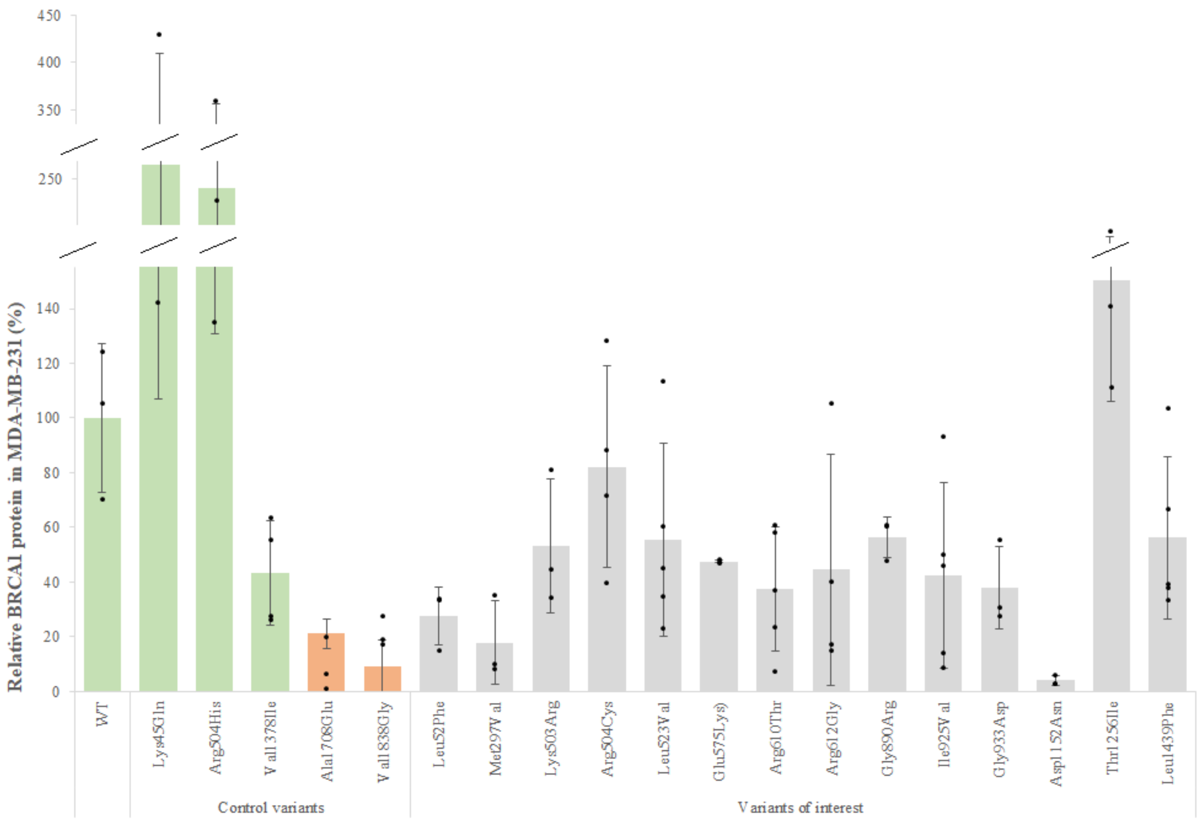

Supplementary Figure S1 - Protein expression levels of BRCA1 variants determined by western blot analysis. A) HEK293FT cells were transiently transfected with BRCA1 WT, known benign and pathogenic control variants and 14 missense BRCA1 VUSs. BRCA1 was detected with anti-BRCA1 antibody. Actin was used as loading control to normalise the corresponding BRCA1 bands. The figure shows images from one representative replicate. B) MDA-MB-231 cells were transiently transfected with BRCA1 WT, known benign and pathogenic control variants and 14 missense BRCA1 VUSs. The black dots represent individual normalised band intensities. Each column represents the mean of three to five

independent replicates (n = 3–5). The benign (green) and pathogenic (orange) control variants are grouped to the left. Error bars represent standard deviation.

A

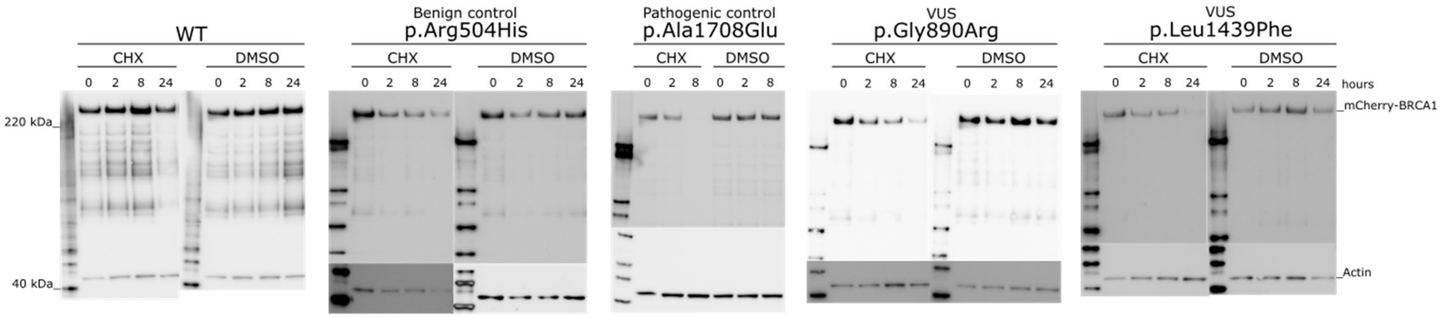

B

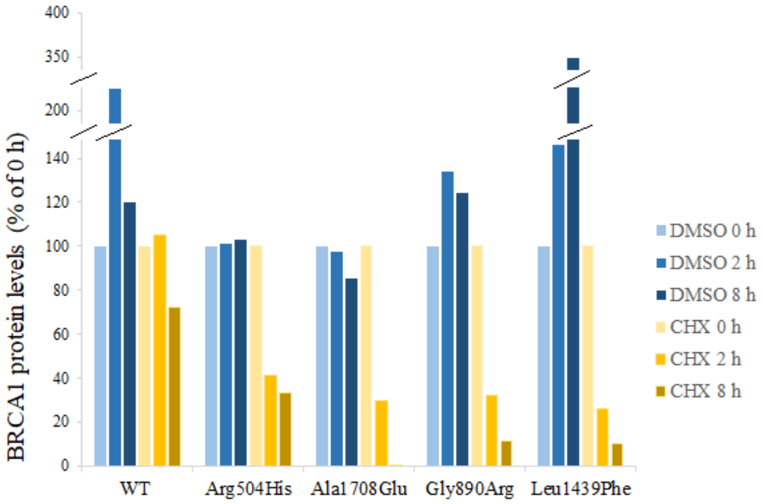

Supplementary Figure S2 – Assessment of protein stability after 0, 2 and 8 hours treatment with cycloheximide or DMSO only. A) The figure shows western blot images for HEK293FT cells transiently transfected with *BRCA1* WT, the two variants showing the highest levels of protein degradation (p.Gly890Arg and p.Leu1439Phe) after 0-8 hours treatment with cycloheximide (CHX), a benign control (p.Arg504His) and a pathogenic control

(p.Ala1708Glu) (results for several controls and seven BRCA1 variants are not shown). Cells were treated with cycloheximide dissolved in DMSO or DMSO only for comparison. Samples were harvested after 0, 2 and 8 hours of treatment followed by western blotting. BRCA1 was detected with anti-BRCA1 antibody. Actin was used as loading control to normalise the corresponding BRCA1 bands. **B)** Western blot bands from three to five biological replicates were quantified in Image Lab software. The normalised protein levels at each time point are shown relative to the amount of each variant at time 0 hours (100%). The graphs illustrate the mean % of all replicates after 0-8 hours treatment with cycloheximide or DMSO only.

A

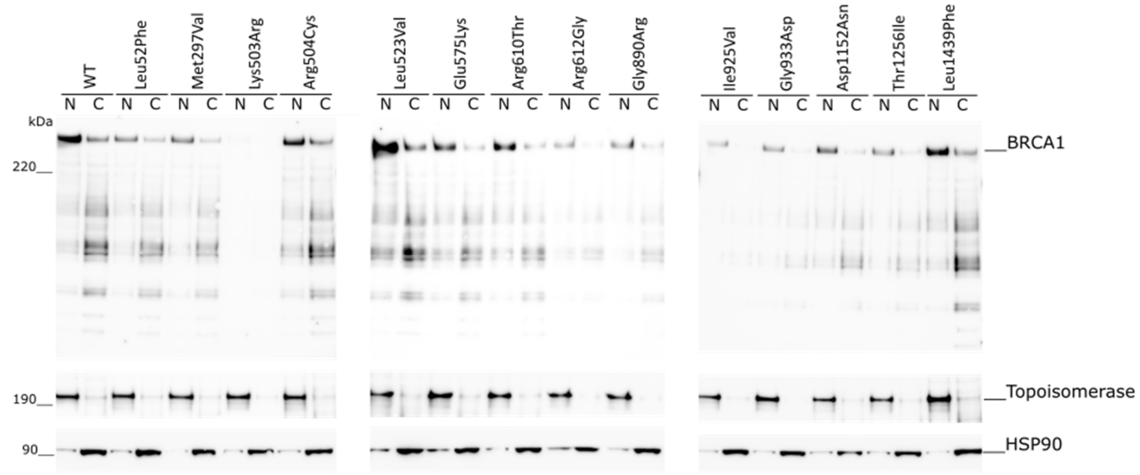

B

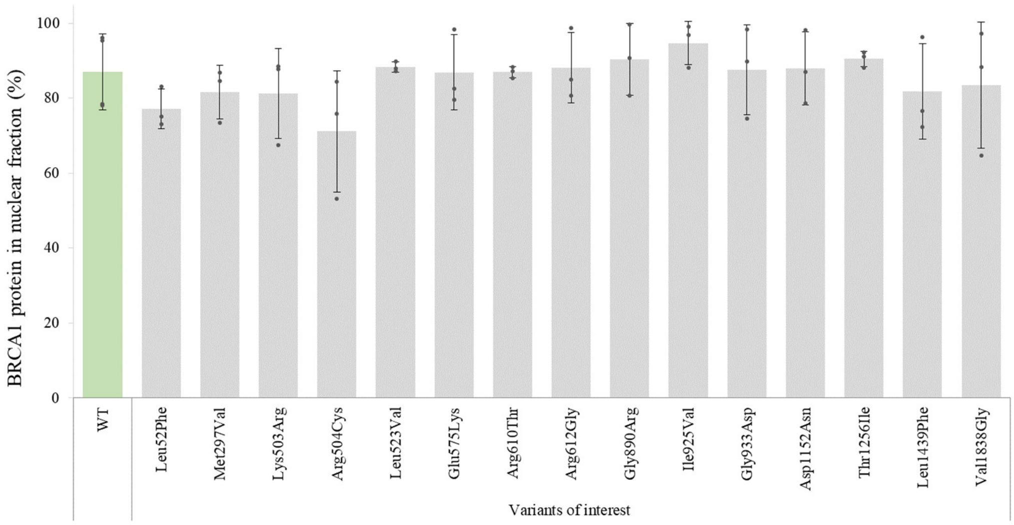

Supplementary Figure S3 – Assessment of nuclear localisation of BRCA1 variants by cellular fractionation assay. A) HEK293FT cells were transiently transfected with *BRCA1* WT and VUSs. After 48 hours, cells were harvested and separated into nuclear and cytosolic fractions and analysed by western blotting. BRCA1 was detected with anti-BRCA1 antibody. Topoisomerase IIα (190 kDa) and HSP 90α/β (90 kDa) were used as nucleus and cytosol markers, respectively. **B)** The signals from each band in the western blot images were used for normalisation using Image Lab software. The columns show the percentage of the total BRCA1 protein variant which is located in the nucleus (mean of three independent replicates). The black dots represent individual normalised band intensities. Each error bar represents the standard deviation.
